# Genome-wide association mapping reveals genes underlying population-level metabolome diversity in a fungal crop pathogen

**DOI:** 10.1186/s12915-022-01422-z

**Published:** 2022-10-08

**Authors:** Nikhil Kumar Singh, Sabina Moser Tralamazza, Leen Nanchira Abraham, Gaétan Glauser, Daniel Croll

**Affiliations:** 1 Laboratory of Evolutionary Genetics, Institute of Biology, University of Neuchâtel, 2000 Neuchâtel, Switzerland; 2 Present Address: Institute of Phytopathology, Christian-Albrecht University of Kiel, 24118 Kiel, Germany; 3 Neuchâtel Platform of Analytical Chemistry, University of Neuchâtel, 2000 Neuchâtel, Switzerland

**Keywords:** Fungal pathogens, *Zymoseptoria tritici*, Whole-genome sequencing, Metabolomics, Metabolite genome-wide association mapping, Specialized metabolites, Metabolite-GWAS

## Abstract

**Background:**

Fungi produce a wide range of specialized metabolites (SMs) involved in biotic interactions. Pathways for the production of SMs are often encoded in clusters of tightly arranged genes identified as biosynthetic gene clusters. Such gene clusters can undergo horizontal gene transfers between species and rapid evolutionary change within species. The acquisition, rearrangement, and deletion of gene clusters can generate significant metabolome diversity. However, the genetic basis underlying variation in SM production remains poorly understood.

**Results:**

Here, we analyzed the metabolite production of a large population of the fungal pathogen of wheat, *Zymoseptoria tritici*. The pathogen causes major yield losses and shows variation in gene clusters. We performed untargeted ultra-high performance liquid chromatography-high resolution mass spectrometry to profile the metabolite diversity among 102 isolates of the same species. We found substantial variation in the abundance of the detected metabolites among isolates. Integrating whole-genome sequencing data, we performed metabolite genome-wide association mapping to identify loci underlying variation in metabolite production (i.e., metabolite-GWAS). We found that significantly associated SNPs reside mostly in coding and gene regulatory regions. Associated genes encode mainly transport and catalytic activities. The metabolite-GWAS identified also a polymorphism in the 3′UTR region of a virulence gene related to metabolite production and showing expression variation.

**Conclusions:**

Taken together, our study provides a significant resource to unravel polymorphism underlying metabolome diversity within a species. Integrating metabolome screens should be feasible for a range of different plant pathogens and help prioritize molecular studies.

**Supplementary Information:**

The online version contains supplementary material available at 10.1186/s12915-022-01422-z.

## Background

Fungi are capable of synthesizing a wide range of compounds including amino acids, pigments, antibiotics, and toxins [1–3]. Such metabolites are typically classified as primary and specialized (or secondary) metabolites. Primary metabolites are essential for growth, development, and reproduction and tend to be conserved across the phylogeny of fungi. Specialized metabolites (SMs) confer benefits in specific ecological niches but are not essential for cell survival [4]. The production of primary metabolites is encoded by a vast array of enzymes involved in metabolic reactions. In contrast, the production of SMs in fungi is often encoded by a small set of enzymes establishing a minimal metabolic pathway. The underlying genes tend to be arranged in close proximity forming a biosynthetic gene cluster (BGCs) including tight regulatory control [5]. SMs play an important role in shaping species interactions with the microbiome. For example, the production of the antibacterial bikaverin, which is induced upon contact with the bacterium *Ralstonia solanacearum*, is conserved in distant fungal pathogens [6]. Besides, SMs are essential for regulating fungal pathogenicity by acting as virulence factors against animal and plant hosts [7]. In the case of *Fusarium graminearum*, the BGC encoding the pathway for the production of the mycotoxin trichothecene is upregulated during colonization of wheat [8] and essential for fungal colonization of kernels. SM can also act as messengers in inter- and intra-species communications between fungi [9, 10]. The high degree of niche specificity of SMs generates diversity in BGCs among closely related species and even within some species [5, 11]. BGCs are also among the most mobile elements in fungal genomes with extensive evidence for horizontal gene transfer and turnover within species [12].

Large-scale fungal genome analyses have revolutionized the discovery of genes underlying the specialized metabolism and rapidly evolving BGCs transferred among species [11, 13, 14]. Genetic changes underlying the production of SM can be pinned down to single nucleotide variation as shown for fumonisin production in *Fusarium* fungi [15]. The evolution of BGCs often involves larger changes including the gain and loss of genes [16, 17]. BGCs are often located in more repetitive regions of the genome including subtelomeres [18]. Furthermore, BGCs in some groups of fungi are mainly regulated by epigenetic changes including chromatin modification [19]. The fact that BGCs encode entire pathways for the production of SM, horizontal gene transfers effectively mean for species to acquire novel ecological functions [20–22]. Horizontal gene transfers and rearrangements may lead to substantial differences within and among closely related species in the content of BGCs and the potential to produce SMs. Among *Aspergillus* fungi, BGCs contribute to shared virulence profiles based on gliotoxin and fumitremorgin production and species-specific virulence, e.g., due to fumagillin [23]. Genetic differences in qualitative and quantitative variation of SM production are hence likely under strong selection. Variation in metabolite production among conspecific isolates can be used for association mapping. Identifying associations between genetic variants in populations with relative levels of metabolite production is performed as metabolite genome-wide association studies (mGWAS) with a primary focus on the plant model *Arabidopsis thaliana* [24]. Recent applications on rice cultivars identified genes underlying flavonoid production [25] and contributions to the phenol-amides metabolic pathway [26]. Metabolite variation was often mapped to a small number of major effect loci [27, 28]. The intra-specific variation in metabolite profiles, high-quality genomic resources, and experimental tractability make fungi attractive models for genome-wide association mapping approaches.

The ascomycete *Zymoseptoria tritici* is a highly polymorphic fungal pathogen of wheat and shows a marked variability in BGCs among members of the species [29–31]. The pathogen causes yield losses of ~ 5–30% depending on environmental conditions [32, 33]. Populations sampled across the world show evidence for the gain and loss of genes constituting BGCs [34]. Comparisons of complete genome assemblies confirmed that BGCs are partly or entirely missing in some isolates [31] raising questions about the functional relevance of the encoded SMs. However, the role of SM in the lifecycle of *Z. tritici* remains poorly understood. A metabolome analysis of the wheat infection process showed that lipid metabolism is the initial energy source during leaf colonization prior to the induction of host cell death [35]. The pathogen upregulates BGCs mostly during the same transition to feeding from dead plant material (i.e., necrotrophic lifestyle) ~10 days after infection [35, 36]. Precursors of melanin were also found in the metabolite profile at this stage [35]. Melanin, which plays an important role in virulence, ultraviolet protection, and anti-microbial resistance in fungi, is one of the best-studied SMs of *Z. tritici.* The locus underlying variation in melanin production was first identified using quantitative trait mapping and then confirmed to be a polyketide synthase gene cluster [37, 38]. Among individual variation in melanin, accumulation is governed by the insertion of a transposable element, which impacts the regulation of the BGC [38]. A recent study illuminated chemical diversity among multiple isolates of the same species and identified possible links to BGC diversity [39]. Population-level metabolomic diversity within the species and the underlying genetic basis remain unknown.

Here, we take advantage of genome-wide association (GWA) mapping using the production of individual metabolites under standardized conditions as trait values. GWA mapping in *Z. tritici* has been used successfully to identify the genetic basis underlying virulence on different wheat cultivars, resistance to fungicides, and temperature adaptation [40–43]. We focused on a single wheat field to establish a panel of 102 isolates showing considerable genetic diversity [43]. We performed untargeted ultra-high performance liquid chromatography-high resolution mass spectrometry on individual fungal cultures growing under sterile conditions. We found considerable variation in metabolite profiles with the majority of metabolites showing abundance variation among isolates. GWA mapping revealed significantly associated loci in proximity to genes encoding functions related to transport and catalytic activity.

## Results

### Species-wide polymorphism in specialized biosynthetic gene clusters

**Fig. 1:**
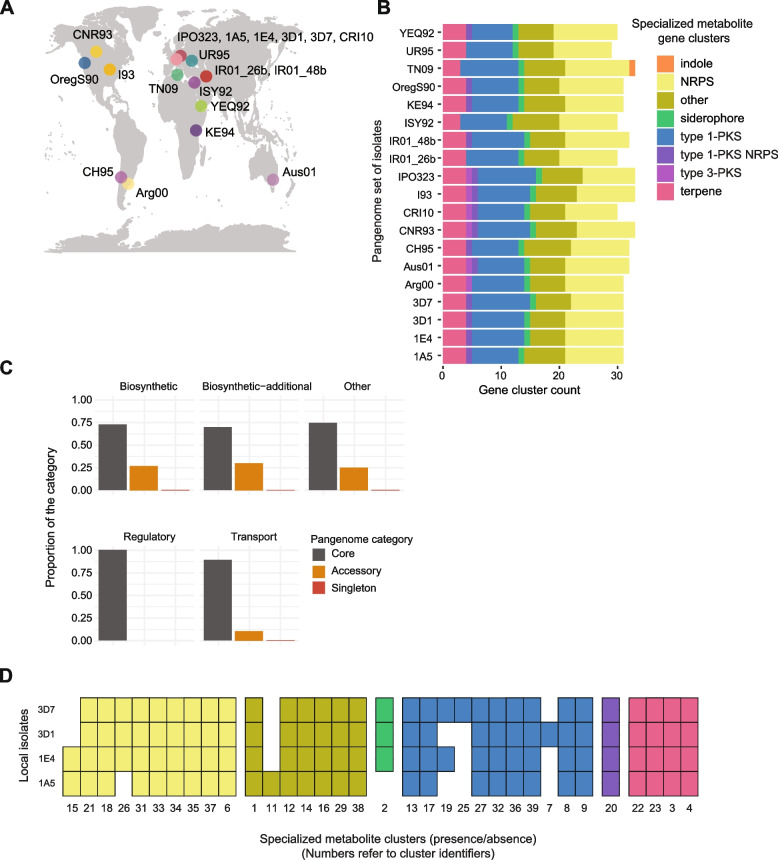
Genetic diversity of specialized metabolite gene clusters in *Zymoseptoria tritici*. **A** World map indicating the isolate names and country of origin. **B** Detected gene clusters in 19 genomes constituting the species pangenome [31]. Colors indicate different categories of specialized metabolite gene clusters depending on the metabolite compound or the core biosynthetic gene: indoles, non-ribosomal peptide synthetase (NRPS), siderophores, polyketide synthase (PKS), and terpenes. **C** The proportions of core, accessory, and singletons specialized metabolite gene clusters in the species pangenome. **D** Presence-absence variation of 34 gene clusters in genomes of four isolates collected in Switzerland

A pangenome based on 19 genomes of *Z. tritici* isolates collected from six continents and 13 different countries (Fig. 1A) [31] was used to retrieve between 29 and 33 BGCs per genome (Fig. 1B). The genomes were assembled without gaps spanning telomere to telomere. Gene models were annotated using transcriptomic datasets for training [31]. Non-ribosomal peptide synthetase (NRPS) and type 1-polyketide synthase were the most abundant gene clusters encoded by the genomes. Approximately 72% of the predicted core biosynthetic genes were shared among isolates and ~24% were accessory (present in 2–18 isolates; Fig. 1C). We also retrieved a singleton core biosynthetic gene found only in an isolate sampled in Tunisia. We found similar proportions for additional biosynthetic genes with ~60% and 30% core and accessory genes, respectively (Fig. 1C). Regulatory genes of gene clusters were all conserved, but only 90% of the transporter genes were conserved (Fig. 1C). BGCs show polymorphism also at the level of single fields. Focusing on BGCs encoded in the genomes of four isolates collected in the same year from nearby wheat fields in central Europe, seven clusters showed presence/absence variation [31] (Fig. 1D).

### Genetic diversity in a single-field mapping population

**Fig. 2:**
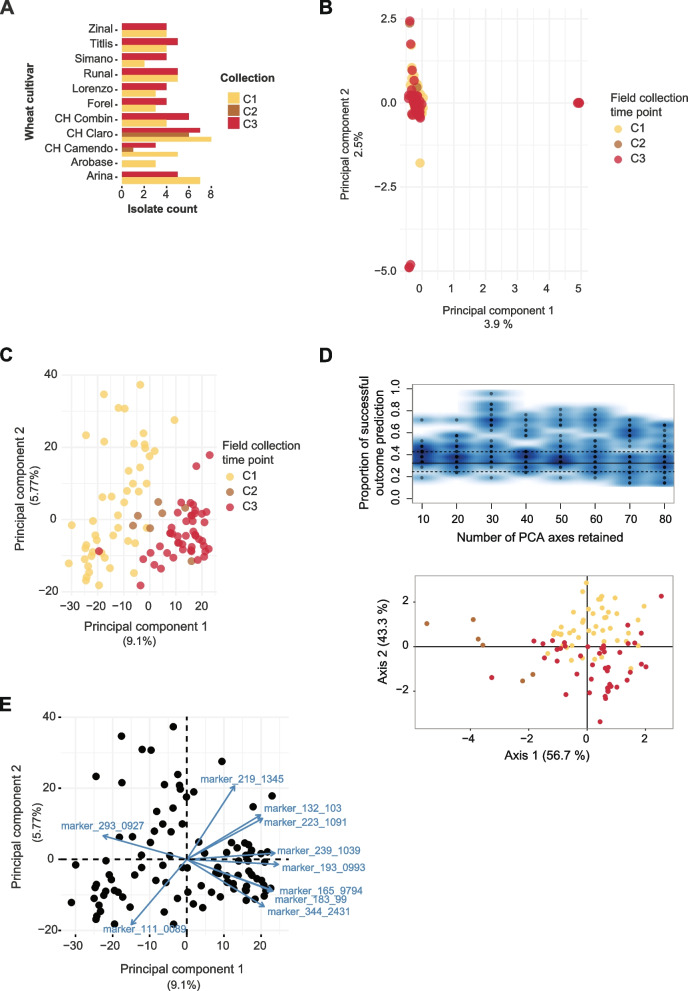
Whole-genome sequencing and untargeted metabolite analyses of 102 *Zymoseptoria tritici* isolates collected from a single field. **A** Number of isolates collected from each of the eleven cultivars at each collection time point (C1–3; early, middle and late in the season; see also [44]). **B** The first two principal components (PC) from a PC analysis of genome-wide SNPs. Isolates are color coded by the collection time point. **C** The first two PCs from a PC analysis of 2633 metabolite markers. Isolates are color coded by the collection time point. **D** Cross-validation for the determination of optimum number of PCs to be retained for the discriminant analysis of principal components (DAPC) and DAPC plot. Isolates are color coded by the collection time point. **E** Bi-plot of 2633 metabolite markers showing the ten metabolite markers contributing most to the differentiation of the metabolite profiles. Metabolite markers are labeled using their respective *m/z* values

To test whether individual isolates differ in the production of metabolites and show heritable genetic variation, we performed a genome-wide metabolite association study (mGWAS). We analyzed whole-genome sequences of 102 strains isolated from a single wheat field during a single growing season [43]. The isolates were collected from 11 genetically different winter wheat cultivars (1–9 isolates per cultivar) from three collection time points (Fig. 2A, Additional file 1: Table S1). At the first collection time point, the wheat seedling was at growth stage (GS) 41 where flag leaves start extending. At the second (GS 75) and third collection (GS 85) time point, plants were fully developed and grains were reaching maturity [45]. The average Illumina sequencing coverage was 21X as previously described [43, 44]. After quality filtering, we retained 504,557 high-confidence single nucleotide polymorphisms (SNPs). To assess whether the mapping population was well-suited for GWAS (i.e., without significant substructure), we first performed a principal component analysis (PCA). The PCA identified a small group of outlier isolates (*n* = 7; Fig. 2B). The percent variance explained was only 3.9% and 2.5% though for principal components 1 and 2, respectively (Fig. 2B). The PCA revealed no meaningful genetic substructure among collection time points or cultivars (Fig. 2B [44];). Furthermore, a PCA performed after removing the seven most differentiated genotypes based on the PCA revealed no meaningful association of genetic differentiation with collection time point or cultivar (Additional file 2: Fig. S1A). We constructed an unrooted phylogenetic network using Splitstree and we found nearly all genotypes to be at similar genetic distances to each other (Additional file 2: Fig. S1B). Finally, we conducted a discriminant analysis of principal components (DAPC) and found that individual principal components made only weak contributions to the overall structure (Additional file 2: Fig. S2) and a single cluster encompassing all genotypes was the most parsimonious grouping (Additional file 2: Fig. S2). The mapping population also included eight clonal groups for a total of 19 isolates [44]. To account for genetic relatedness among genotypes, we included relatedness as a random factor for association mapping (i.e., mixed linear model).

### Untargeted metabolite profiling at the population level

We performed an untargeted metabolite profiling of all 102 isolates using ultra-high-performance liquid chromatography high-resolution mass spectrometry (UHPLC-HRMS). The analyzed metabolites were extracted from blastospores after culture washing. After excluding highly hydrophilic (e.g*.*, sugars, amino acids) and hydrophobic (e.g., lipids) molecules based on retention times, we retained 2633 metabolite marker peaks (Additional file 1: Table S2). Using relative abundance across all peaks, we performed a principal component analysis (Fig. 2C). Interestingly, the metabolite profiles of different isolates clustered based on their field collection time point (Fig. 2C). This contrasts with the genome-wide differentiation at neutral markers (Fig. 2B). Removing the seven most differentiated genotypes from the PCA had no meaningful impact on the grouping of metabolite profiles by collection time point (Additional file 2: Fig. S1A) or analyzing exclusively SNPs within BGC genes (Additional file 2: Fig. S3A). We further investigated the possibility of the metabolite profiles being explained by genome-wide genetic differentiation of the collected isolates. For this, we performed cross-validation to predict the best number of principal components to cluster genotypes based on the collection time point (Fig. 2D). The DAPC analysis showed that ~30 principal components were optimal to cluster according to time point, consistent with a weak association of genome-wide genetic structure and metabolome profiles (Fig. 2D). We analyzed the ten most important metabolite markers contributing to the differentiation in the PCA (Fig. 2E). Variation in five out of ten of these metabolite markers was significantly associated to individual SNPs in the mGWAS (see below; Additional file 1: Table S3). We performed a down-sampling analysis to identify the proportion of metabolite markers detected in isolates. We found approximately equal proportions of metabolite markers detected in 75%, 50%, and 25% of the total isolates (Additional file 2: Fig. S3B).

### Metabolite-GWAS based on variation in metabolite abundance

**Fig. 3:**
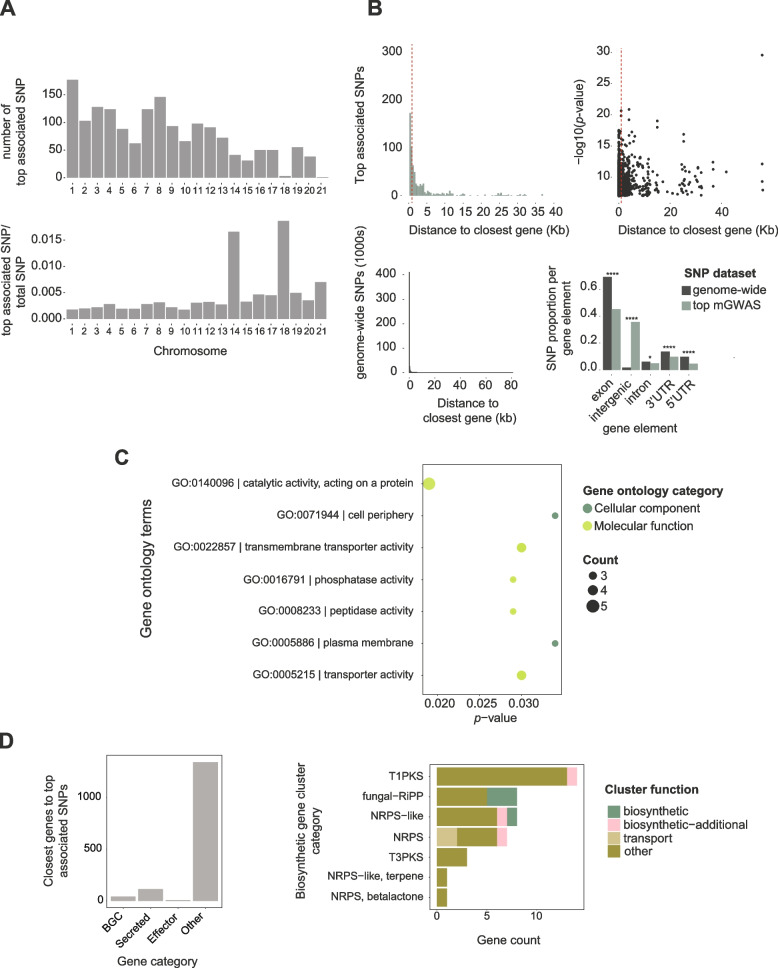
Metabolite genome-wide association studies (mGWAS) and candidate gene functions. **A** Number of the most significantly associated single nucleotide polymorphisms (SNP) and proportion of significantly associated SNPs. **B** Distance to the closest gene and association −log10(*p*) values of the most significantly associated SNP for each metabolite marker. The dotted red line delimits 75% of the SNPs. Number of genome-wide SNP closest to gene. Proportion of SNPs found per gene element across the genome and for the significantly SNPs. **C** Gene ontology term enrichment analysis of functions encoded by the closest genes. **D** Number of the most significantly associated SNP to the closest gene based on gene type, biosynthetic gene cluster class and function. *, **** : *p-*value <0.05, <0.00001, respectively

Variation in relative abundance among isolates may have a genetic basis (Fig. 2C). We performed mGWAS on relative metabolite marker intensities based on mixed linear models taking genetic relatedness into account. We first evaluated the most significant associations based on *p*-values for each of the 2388 untargeted metabolite markers showing distinct *m/z* peaks. After filtering association *p*-values, 68.8% (1644 out of 2388) of untargeted metabolites showed at least one significant SNP association (Additional file 1: Table S3). Overall, we found similar proportions of associated SNPs on core (*n* = 13) and accessory chromosomes (*n* = 8). Accessory chromosomes 14 and 18 showed high proportions of associated SNPs (Fig. 3A). A total of 21 associations were found on chromosome 14 in close proximity to the telomere and ~25 kb from the closest gene (Additional file 1: Table S3). Chromosome 14 contains large repetitive regions stemming from a recent insertion. The repetitiveness of accessory chromosome sequences leads to few reliable SNP calls outside of genes. Hence, the high proportion of associated SNPs on chromosome 14 (and 18) are most likely explained by low overall number of called SNPs rather than a biological reason.

We analyzed the closest gene to the most significantly associated SNP for each metabolite marker (Fig. 3B, Additional file 1: Table S3). Overall, 75% of these most significantly associated SNPs were found at a maximum distance of 915 bp to the closest gene and 50% within 18 bp (Fig. 3B). Genome-wide SNPs are for a large majority (75%) within coding sequences. This is consistent with the gene-dense genome organization, the average distance between genes of ~1 kb [46], and challenges in calling SNPs in repetitive regions [44]. The average intergenic distance also matches the distance at which linkage disequilibrium decays on chromosomes (*r*^2^ < 0.2 within 500–1500 bp) [44]. The abundance of genome-wide SNPs being found within coding sequences is also partially explained by the difficulty of obtaining accurate SNP genotyping calls far from genes. Interestingly, the most significantly associated SNPs were further away from genes than random SNPs and highly enriched for intergenic regions (*p <* 0.00001). This suggests that such variants tend to be linked to regulatory variation. For the remainder, we focused only on the most significantly associated SNPs for each metabolite marker falling within 5 kb of a gene (Additional file 1: Table S3, *n* = 1508).

We performed gene ontology (GO) term enrichment analyses of the protein functions encoded by genes nearest to the most significantly associated SNP (Additional file 1: Table S4). We found that 27% of all nearby genes were assigned GO terms in contrast with 49% for all genes in the genome (Additional file 1: Table S3). We found the strongest enrichment for membrane transport functions and catalytic activity (Fig. 3C, Additional file 1: Table S4). Transporters play key roles in metabolic pathways [11] and often underpin niche adaptation [47].

### Associations of metabolite-GWAS loci with gene clusters

Due to the large number of metabolic traits analyzed (*n* = 2388), we focused on the closest gene to the most significantly associated SNP of each trait (i*.*e., metabolite) to prioritize the most robust metabolic pathway associations. We identified 124 genes encoding functionally predicted proteins including seven proteins characterized as putative effectors (Fig. 3D). We additionally found 42 genes involved in specialized biosynthetic gene clusters (BGC) covering 19 out of the 39 known BGC in the species (Fig. 1B). BGCs of the polyketide, fungal-RiPP, and NRPS classes showed the most associations (Fig. 3D). Core biosynthetic genes accounted for five associations, while the largest number of associations (*n* = 33) were of unknown category. Next, we focused on BGC candidate gene clusters due to their well-established role in fungal metabolism [4]. We manually curated promising targets based on metabolite peak quality, relative marker intensity variation among genotypes, and the proximity of SNPs to gene distance (<500 bp) focusing on SNP most likely residing in regulatory and UTR regions.

**Fig. 4:**
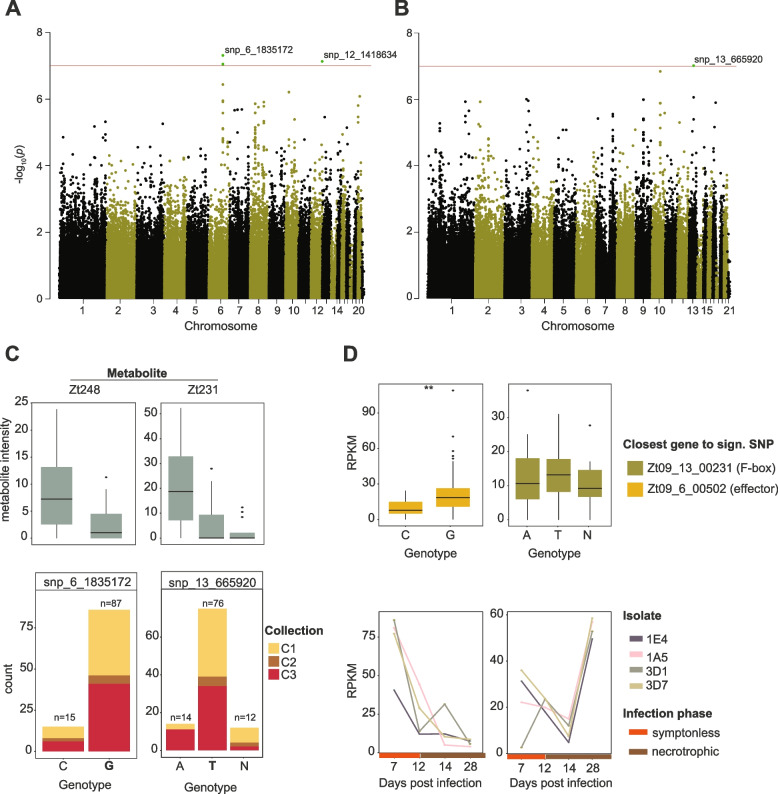
Analyses of key loci identified by the metabolome-GWAS. **A** Manhattan plots of genome-wide association mapping performed for the metabolite Zt248 and **B** the metabolite Zt231 in a *Z. tritici* single-field population (*n* = 102 isolates). The red line refers to the Bonferroni threshold (*α* < 0.05). **C** Relative metabolite intensity and isolate counts based on genotypes (N: unassigned genotype). Colors refer to field collection time point of the isolates. **D** Expression of the closest genes to the focal SNP under culture conditions (same isolates as mapping population; upper box) and of four different isolates collected nearby over the course of an experimental wheat infection (lower box)

**Fig. 5:**
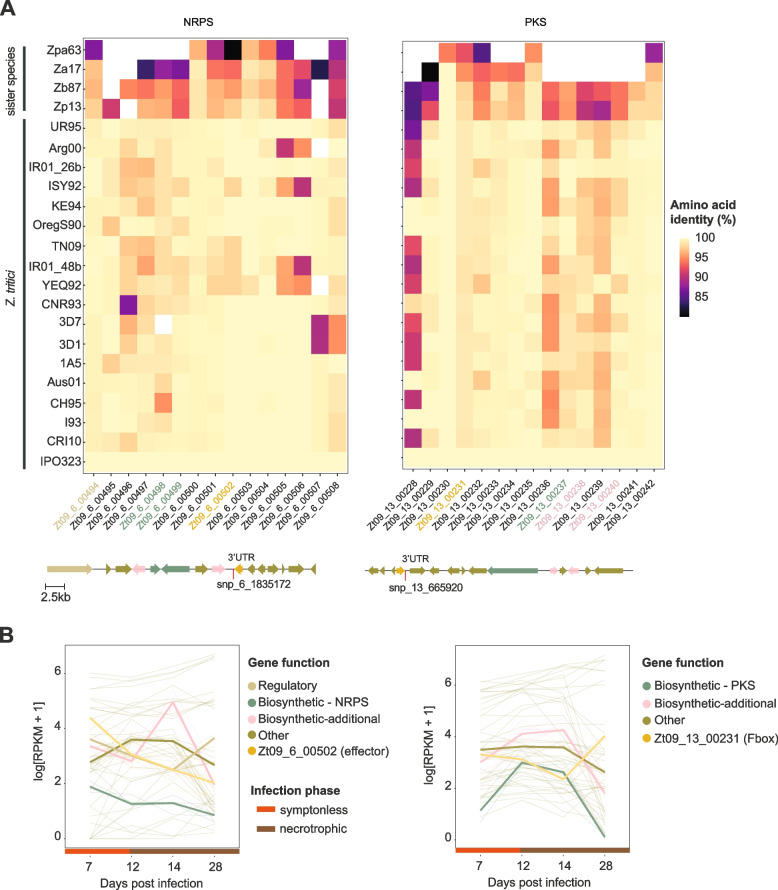
Conservation of the NRPS and PKS biosynthetic gene clusters in the *Z. tritici* pangenome and sister species. **A** Conservation of the effector Zt_06_00502 gene in the NRPS gene cluster and the F-box gene Zt_13_00231 in the PKS gene cluster. Amino acid identity compared to the gene cluster in the *Z. tritici* reference genome IPO323. Arrows below the heatmap correspond to genes locations and SNP positions in the reference genome. **B** Mean expression of the NRPS and PKS gene clusters based on four isolates collected in a nearby field (3D1, 3D7, 1A5 and 1E4). Colors identify gene functions. NRPS: nonribosomal peptide-synthetase, PKS: polyketide synthase. Sister species isolates Zp13: *Z. pseudotritici*, Zpa63: *Z. passerinii*, Zb87: *Z. brevis*, Za17: *Z. ardabiliae*

Our first focus was on the biallelic SNP at 1,835,172 bp on chromosome 6 showing a significant association with the metabolite Zt248 (m/z of 248.1323 and retention time of 3.52 min; Fig. 4A). We found that 14% of the population carried the alternative C allele associated with higher metabolite production (Fig. 4C). We found no association of the C allele with the field collection time point (Fig. 4C). The SNP is 180-bp downstream (3′UTR) of a functionally predicted effector gene Zt09_6_00502 (Additional file 1: Table S3). Interestingly, Zt09_6_00502 was identified as a component of a NRPS gene cluster (cluster 18; Fig. 1D)*.* The gene cluster is 35kb in size and predicted to carry 15 genes including two biosynthetic core genes and a regulatory gene (Fig. 5A). The gene cluster is conserved within the species and shared between three closely related sister species (Fig. 5A). Based on RNA-seq analyses of the same isolates growing in a minimum medium liquid culture, we found a significant association between transcription of the gene Zt09_6_00502 and the genotypes at the focal mGWAS SNP (*p-value* < 0.001; Fig. 4D). Hence, the locus may mediate metabolite production through variation in gene expression. We found that the biosynthetic core genes share a similar transcription profile as the effector gene indicating co-regulation during the wheat infection cycle. Furthermore, the predicted effector is primarily expressed during the initial phase of the infection suggesting contributions to the onset of the disease (Fig. 4D). We analyzed data on controlled infections based on the same set of mGWAS isolates and found that the isolates carrying the alternative genotype caused more lesions (median of 71% of leaf area covered by lesion) in contrast to strains carrying the reference genotype (median of 57% of leaf area covered by lesions; Additional file 2: Fig. S4).

We performed a multiple sequence alignment of the 3′UTR region containing the significantly associated SNPs using genomic sequences from species across the *Zymoseptoria* genus (Additional file 2: Fig. S5A). The phylogenetic context of the 3′UTR indicates that the alternative allele is derived in *Z. tritici*. Interestingly, variants in the gene sequence of Zt09_6_00502 are consistent with geographic differentiation of the species. In contrast, variants in the 3′UTR region reveal haplotypes shared between the Swiss field population (ST16CH_1A27 and ST16CH_1M28) and geographically distant isolates from Yemen (Yeq92) and Tunisia (TN09; Additional file 2: Fig. S5B, C). Pairwise linkage disequilibrium analyses of the BGC showed higher values (*r*^2^ < 0.2 at ~6000 bp) compared to genome-wide expectations (*r*^2^ < 0.2 at ~500bp for core chromosome [44]) (Additional file 2: Fig. S5 D, E) consistent with recent selection and/or epistasis to maintain regulatory functions of the BGC.

We investigated a second locus in detail located at 665,920 bp on chromosome 13 with a significant association with metabolite Zt231 (m/z 231.171 and retention time of 2.24 min; Fig. 4B). Isolates carrying the alternative allele A showed higher metabolic intensity relative to isolates carrying the reference allele T (Fig. 4C). The SNP is located 255 bp downstream (3′UTR) of the F-box gene Zt09_13_00231. The encoded F-box domain (positions 55–99) overlaps with a leucine-rich region (LRR domain; amino acid positions 42–374; Additional file 2: Fig. S6). Expression analyses of Zt09_13_00231 revealed no significant association with the genotypes identified through mGWAS under culture conditions (Fig. 4D). However, we identified an expression quantitative trait locus (eQTL) mapped using the same single-field population at the same mGWAS locus (snp_13_664471; Abraham et al. DOI pending). The transcription of the F-box Zt09_13_00231 gene was upregulated at the beginning of the infection (7 dpi) followed by a decrease during the transition to the necrotrophic phase (12–14 dpi) and a final increase at 28 dpi late in the infection (Fig. 4D). The gene Zt09_13_00231 is predicted to encode a component of the PKS gene cluster 32 (Fig. 1B). The cluster is conserved within *Z. tritici* and the sister species *Zymoseptoria brevis* (Fig. 5A). The gene cluster includes a PKS core gene and two additional biosynthetic genes. Interestingly, we found that the transcription of the BGC is largely antagonistic to the transcription of the F-box gene (Zt09_00231) suggesting a negative feedback mechanism during infection (Fig. 5B). Experimental infections of wheat with a subset of the field population (*n* = 76) showed a trend of higher pycnidia production of the isolates carrying the alternative genotype associated with higher metabolite production (Additional file 2: Fig. S4).

### Chemical characterization of associated metabolites

We analyzed the two metabolites Zt248 and Zt231 further using chemical and natural product databases. The prediction based on the software Canopus [48] classified the metabolite Zt248 (C_11_H_21_NO_3_S) with 90% confidence as a cysteine derivative. The PubChem database classified Zt248 as an N-Acetyl-S-(2-ethylbutyl)-L-cysteine. The metabolite Zt231 (C_11_H_22_N_2_O_3_) produced fragments at m/z 86.097 typical for leucine and isoleucine moieties. A natural product database search retrieved N-Valyl-Leucine as the most likely compound for Zt231. Our analysis using MS/MS showed three peaks at 2.15, 2.24, and 2.37, corresponding to the D-l, L-D, and L-L isoforms (Additional file 2: Fig. S7). We searched for possible analogues and found that confluenine A produced by the basidiomycete *Albatrellus confluens* [49] shares the same molecular formula. The underlying polyketide BGC shares no homology among known fungi though. According to a CFM-ID in silico fragmentation confluenine A should fragment into m/z 85.065 and metabolite Zt231 should fragment to m/z 85.02 and 86.09.

## Discussion

We used untargeted metabolite profiling and genome-wide association mapping to identify candidate loci underlying the production of metabolites. We found that a single, highly diverse field population of *Z. tritici* harbors substantial variation in the production of individual compounds. We performed association mapping based on standardized metabolite peak intensities as a phenotypic trait and identified significantly associated loci in close proximity to regulatory regions encoding virulence factors and biosynthetic gene clusters.

We identified highly variable metabolite profiles among isolates from a single field population. Given the standardized conditions, genetic factors are likely contributing to the observed metabolome diversity. The analyzed population is genetically highly diverse [44]. Surprisingly, overall metabolite profiles clustered according to the isolate collection time point over the growing season (i.e., C1-C3 spread over several weeks). This is in contrast to the genetic differentiation of the same isolates where no clustering is evident based on collection time [44]. A DAPC provided consistent findings in that collection time point does not reasonably explain genome-wide differentiation. However, this does not preclude that individual loci could be strongly differentiated among collection time points. A possible explanation for the consistent shifts of the metabolome over the course of the growing season is the selection for isolates expressing a specific set of metabolites. Such candidate loci could be discovered using genetic differentiation scans along the genome; however, the sample size is limited for sufficiently powerful tests and a series of confounding environmental factors would have to be accounted for. Focusing on genetic differentiation at SNPs segregating in gene clusters, we find no meaningful differentiation by time point consistent with no overall selection BGC polymorphism. More expansive datasets would be required to distinguish selection signatures metabolite loci and to distinguish selection from neutral processes impacting differentiation over time.

One possible environmental factor driving selection is the two fungicide treatments applied midway between the collections C1 and C2 [44]. Additional shifts in phenotypic traits were also apparent for pathogen reproduction (i.e., pycnidia formation) on wheat leaves with isolates collected later in the season showing higher reproductive output under controlled conditions [43, 44]. Shifts in aggressiveness were also previously observed in multi-year studies of *Z. tritici* over years, cultivars and leaves on individual plants [50]. Identifying the chemical structure of the compounds contributing most strongly to shifts in the metabolome over the course of the growing season will provide insights into the adaptive nature of such changes.

Variation in metabolite abundance among individuals can be used to identify candidate genes underlying the regulation of metabolite production. We found a strong enrichment of intergenic regions contributing to metabolite production suggesting *cis*-regulation (i.e., promoter regions) playing a role in shaping within species metabolome diversity. We identified genetic polymorphisms close to genes encoding a broad range of functions with an enrichment of transporter and catalytic functions. Such transporters include major facilitator superfamily (MFS) transporters known to modulate fungicide and stress tolerance [51–53]. MFS transporters are also involved in the secretion of phytotoxins during infection and, hence, can contribute to virulence [54–56].

The studied species harbors a high degree of polymorphism in BGCs with likely consequences for the production of specialized metabolites. The mGWAS captured 41 BGC-related associations affecting approximately half of all known BGCs of the species*.* Standing variation for the production of SM elevates the evolutionary potential of *Z. tritici*, because populations could respond rapidly to selection for or against the production of specific metabolites. Intra-species variation in BGCs and SM production have been observed in a range of fungi including the rice blast pathogen *Pyricularia oryzae*, where a gain of virulence was linked to the duplication of a hybrid PKS-NRPS cluster [53]. *Aspergillus* species show also significant intra- and interspecific variation in BGCs and the potential to produce specific SM with consequences for niche adaptation [57].

We investigated in detail two associations linking a SNP segregating in a BGC with clear metabolite variation. The first SNP was found in the candidate effector gene Zt09_00502, which is a component of the NRPS gene cluster 18 upregulated during the initial phase of infection. Pathogens secrete effectors to manipulate the host immune responses and physiology to its advantage [58]. The production of the metabolite is also associated with the expression of the candidate effector. Higher aggressiveness of the isolates was weakly associated with higher levels of the metabolite. Establishing causal links will require experimental approaches such as allelic replacements or silencing, since the genetic background and epigenetic factors may also influence the phenotypic trait. In fungi, BGCs are often regulated by epigenetic factors such as histone methylation [19]; however, there is no indication that *Z. tritici* BGCs show such an association as well [39]. The conservation of the gene cluster including the effector suggests that the cluster has gained its original function prior to the specialization of the pathogen on wheat. However, the recently arisen adaptive mutation associated with the metabolite production could be an evolutionary response of the pathogen to gain an advantage on wheat.

The second investigated metabolite association was within the UTR region of the gene Zt09_13_00231 encoding an F-box protein, which generally binds and transports proteins to be discarded by the cell [59]. The encoded protein contains domains consistent with a typical F-box FBLX family protein shown to be involved in conidiation, carbon intake, and virulence regulation in fungi [60, 61]. Deletion of the gene encoding a homologous F-box MoGrr1 in the fungal pathogen *P. oryzae* resulted in reduced lesion sizes on rice [62]. The dipeptide Zt09231 and the encoded F-box protein possibly play a regulatory role for the PKS biosynthetic gene cluster. Dipeptides were implicated in biosynthetic pathway regulation at least in some bacteria [63]. Chemical structure predictions and database searches revealed that the metabolite Zt231 associated with the F-box gene shares a structure matching confluenine A. This metabolite has antimicrobial activities and is produced by the basidiomycete *A. confluens* [49]. Convergent molecular structures may be an indicator of similar functionality; however, detailed chemical analyses and interaction studies on the plant are needed.

## Conclusions

Our study highlights the power of association mapping to identify candidate loci underlying metabolite diversity and associated with the host infection process. Previously, mGWAS was limited to plant models, but our findings illustrate how applications to plant pathogenic fungi can efficiently produce candidate lists. A key requirement is access to large, experimentally tractable isolate collections. High degrees of recombination and rapid decay of linkage disequilibrium are further requirements for the successful application of association mapping approaches [64]. Establishing metabolome-wide profiles for a range of pathogens under variable conditions will complement analyses focusing on proteaceous effectors.

## Methods

### Field collection and storage

We collected *Z. tritici* isolates from the Field Phenotyping Platform (FIP) site of the ETH Zürich, Switzerland (Eschikon, coordinates 47.449° N, 8.682° E) [65]. We analyzed a total of 102 isolates collected during the 2016 growing season from 11 winter wheat cultivars, which are commonly grown in Switzerland [66]. We analyzed isolates originating from three collection time points over the season (Additional file 1: Table S1). The first collection (*n* = 48) was established in May when wheat plants were in growth stage (GS) 41. The second (*n* = 7) and third collections (*n* = 47) were established when the plants were in GS 75 and GS 85, respectively. After sampling, spores of each isolate were stored in either 50% glycerol or anhydrous silica gel at −80 °C. Additional information regarding the sampling scheme and genetic diversity of the collection is described in [44].

### Culture preparation and metabolite extraction

Isolates were revived from glycerol stock by adding 50 μl fungal stock solution to a 50-ml conical flask containing 35 ml liquid YSB (yeast-sucrose broth) medium. The inoculated flasks were incubated in the dark at 18°C and 160 rpm on a shaker-incubator. After 8 days of incubation, the cultures were passed through four layers of meshed cheesecloth and washed thrice with sterile milli-Q water to remove media traces. Spores were then lyophilized and metabolites extracted by resuspending the spores (~80 mg) in 1 ml of HPLC-grade methanol. The extract was centrifuged at 15,000 rpm for 5 min to pellet down debris before retrieving the supernatant. The last step was repeated until a clear supernatant was recovered.

### Whole-genome sequencing and variant calling

Approximately 100 mg of lyophilized spores was used to extract high-quality genomic DNA with the Qiagen DNeasy Plant Mini Kit according to the manufacturer’s protocol. We sequenced paired-end reads of 100 bp each with an insert size of ~550 bp on the Illumina HiSeq 4000 platform. Raw reads are available on the NCBI Sequence Read Archive under the BioProject PRJNA596434 [67, 68]. Illumina sequences were quality-checked using FastQC v. 0.11.9 [69].. Sequencing reads were then screened for adapter sequences and quality trimmed using Trimmomatic v. 0.39 [70] using the following settings: illuminaclip=TruSeq3-PE.fa:2:30:10, leading=10, trailing=10, sliding-window=5:10, and minlen=50. Trimmed sequencing reads were aligned to the reference genome IPO323 [46] available from Ensembl Fungi (https://fungi.ensembl.org/Zymoseptoria_tritici/Info/Index) and the mitochondrial genome (European Nucleotide Archive accession EU090238.1) using Bowtie2 v. 2.4.1 [71]. Multi-sample joint variant calling was performed using the HaplotypeCaller and GenotypeGVCF tools of the GATK package v. 4.0.1.2 [72]. We retained only SNP variants (excluding indels) and proceeded to hard filtering using the GATK VariantFiltration tool based on the following cutoffs: QD < 5.0; QUAL < 1000.0; MQ < 20.0; −2 > ReadPosRankSum > 2.0; −2 > MQRankSum > 2.0; −2 > BaseQRankSum > 2.0. Next, we filtered for locus level genotyping rate (>50%) and minor allele count (MAC) of 1 using VCFtools v. 0.1.15 [73]. Additional sequencing and variant call statistics are available from [44].

#### Population structure analyses

We reduce the SNP dataset using vcftools –thin [73] with a 1000-bp window to randomly select a single SNP per window in order to reduce linkage disequilibrium among loci and computational demands. To investigate population structure of the mapping population, we first performed principal component (PCA) analyses. We processed the filtered SNPs using the R packages vcfR v. 1.8.0 [74] and PCs were calculated using the function dudi.pca() of the R package ade4 v. 1.7-16 [75]. The data was visualized using ggplot2 v. 3.1.0 [76]. Dudi.pca()uses the same model as the base prcomp function in R. We generated an unrooted phylogenetic network using SplitsTree v4.14.6 using uncorrected *p* distances [77]. We performed a pairwise homoplasy index (Phi) test for recombination using SplitsTree v4.14.6 [77]. The cross-validation and discriminant analysis of principal components (DAPC) was performed using adegenet v2.1.7 R package [78]. All file format conversions were performed using PGDSpider v2.1.1.5 [79].

### Functional annotation of genes

We analyzed gene models predicted for the reference-quality genomes assembled for the pangenome of the species with models trained using RNA-seq datasets [31]. Protein functions were predicted using InterProScan v 5.31-70.0 [80]. Putative effectors were identified among the set of secreted proteins using EffectorP v 2.0 [81]. BGCs were predicted using antiSMASH 4.0 [82]. Genes included in a predicted cluster were annotated as “biosynthetic,” “biosynthetic-additional,” “transport,” “regulatory,” or “other” (i.e., not matching any other category).

### Untargeted metabolite profiling using UPLC-HRMS

Metabolome analyses were carried out by UHPLC-HRMS using an Acquity UPLC coupled to a Synapt G2 QTOF mass spectrometer (Waters). An Acquity UPLC HSS T3 column (100x2.1mm, 1.8 μm; Waters) was employed at a flow rate of 500 μl/min and maintained at a temperature of 40°C. The following gradient with 0.05% formic acid in water as mobile phase A and 0.05% formic acid in acetonitrile as mobile phase B was applied: 0–100 % B in 10 min, holding at 100% B for 2.0 min, re-equilibration at 0% B for 3.0 min. The injection volume was 2.5 μl. The QTOF was operated in electrospray negative mode using data-independent acquisition (DIA) alternating between two acquisition functions, one at low and another at high fragmentation energies. Mass spectrometric parameters were as follows: mass range 50–1200 Da, scan time 0.2 s, source temperature 120°C, capillary voltage −2.5 kV, cone voltage −25V, desolvation gas flow and temperature 900 L/h and 400°C respectively, cone gas flow 20 L/h, collision energy 4 eV (low energy acquisition function) or 15–50 eV (high energy acquisition function). A 500 ng/ml solution of the synthetic peptide leucine-enkephaline in water:acetonitrile:formic acid (50:50:0.1) was infused constantly into the mass spectrometer as internal reference to ensure accurate mass measurements (<2ppm). Data was recorded by Masslynx v.4.1. Marker detection was performed using Markerlynx XS (Waters) with the following parameters: initial and final retention time 1.5 and 10.0 min, mass range 85–1200 Da, mass window 0.02 Da, retention time window 0.08 min, intensity threshold 500 counts, automatic peak width and peak-to-peak baseline noise calculation, deisotoping applied. Data was mean-centered and Pareto scaled before applying multivariate analysis.

### Genome-wide association mapping and linkage disequilibrium analyses

We performed GWAS based on mixed linear models accounting for degrees of kinship among genotypes (MLM+K). The kinship matrix was computed using the scaled identity-by-state (IBS) algorithm implemented in TASSEL v. 20201114 [83]. We included the kinship matrix as a random effect in the mixed linear models for association mapping using TASSEL. Accounting for kinship performs sufficiently well to control for genetic substructure in the mapping population [43]. Untransformed relative abundance values for each peak were used as trait values for association mapping. Outcomes were visualized using the R package qqman v. 0.1.4 [84]. We filtered association *p*-values based on the Bonferroni threshold at alpha = 0.05. Per metabolite, we selected the closest gene to the most significant associated SNP using the “closest” command in bedtools v. 2.29.0 [85]. Linkage disequilibrium was performed using PLINK v.1.0 [86] and visualized with the R package LDheatmap 1.0 [87]. GO term enrichment analyses were performed using the Fisher’s exact test based on gene counts with the topGO R package [88] and plotted using the GOplot R package [89]. To investigate mGWAS-associated loci in close proximity to BGCs, we filtered for SNPs at a minimum distance of 500 bp to cluster edges and analyzed metabolites exhibiting minimal m/z peak intensity to chemical prediction quality (intensity above >5).

### RNA-seq analyses

To investigate loci during the fungal life cycle, we used public RNA-seq dataset (NCBI Short Read Archive accession number SRP077418) of four isolates included in the pangenome dataset (3D1, 3D7, 1E4, and 1A5). The isolates were inoculated on wheat plants to monitor transcription levels during the infection process at four time points (7, 12, 14, and 28 days after infection) [90]. For transcriptional profiling of the field population, we cultured the same isolates (*n* = 102) in a Vogel Minimal N Medium [91] where ammonium nitrate was replaced with potassium nitrate and ammonium phosphate. The medium contained no sucrose and agarose to induce hyphal growth. Total RNA was isolated from the filtered mycelium after 10–15 days using the NucleoSpin® RNA Plant and Fungi kit. For all analyses, the Illumina raw reads were trimmed and filtered for adapter contamination using Trimmomatic v. 0.32 with parameters: ILLUMINACLIP:Trueseq3_PE.fa:2:30:10 LEADING:3 TRAILING:3 SLIDINGWINDOW:4:15 MINLEN:36 [70]. Filtered reads were aligned using Hisat2 v. 2.0.4 with default parameters [92] to the *Z. tritici* reference genome (IPO323). Mapped transcripts were quantified using HTSeq-count [93]. Read counts were normalized based on the trimmed mean of M-values (TMM) method using the calcNormFactors option. To account for gene length, we calculated reads per kilobase per million mapped reads (RPKM) values using the R package edgeR [94].

## Supplementary Information

**Additional file 1: Table S1.** *Zymoseptoria tritici* field collection information including collection time point (C1-3), cultivar of origin, field plot. **Table S2.** Relative intensities of 2633 metabolite markers. The isolate name corresponding to sample identifiers are detailed in Table S1. **Table S3.** List of metabolite-GWAS top significant SNPs and distance to closest gene. **Table S4.** Gene function enrichment based on m-GWAS closest genes to top significant SNPs.

**Additional file 2: Figure S1.** A) The first two principal components (PC) from a PC analysis of genome-wide SNPs after removal of outliers (n=7). Isolates are color coded by the collection time point and wheat cultivar. B) SplitsTree phylogenetic network constructed from genome-wide single nucleotide polymorphism (SNP) data. **Figure S2.** Discriminant analysis of principal components (DAPC) results from the genome-wide SNP dataset. Cumulated variance explained by the eigenvalues of the PCs and scatter plot of the Bayesian Information Criterion (BIC) values. The lowest BIC value indicates the most parsimonious number of clusters. **Figure S3.** A) The first two principal components (PC) from a PC analysis of SNPs closest to biosynthetic gene clusters BGC. Isolates are color coded by the collection time point and wheat cultivar. B) Down sampling analysis to identify the proportion of 2633 metabolite markers detected in mapping population. **Figure S4.** Percentage leaf area covered by lesions and pycnidia during wheat infection for subset of *Z. tritici* strains included in the metabolome-GWAS analysis (*n* = 76). The isolates are grouped by their genotype at significant metabolome GWAS SNPs. **Figure S5.** Evolutionary history of the putative effector gene *Zt09_00502*. A) Alignment of the 3’UTR region of the gene. The box refers to the haplotypes found in the single Swiss field population. B) Phylogenetic tree of the 3’UTR region and (C) of the gene sequence. Names in bold black, blue and grey refer to the reference genomes of the species (IPO), isolates included in the metabolite GWAS and sister species, respectively. The phylogenetic tree was inferred by using maximum likelihood and the Tamura-Nei model. D) Pairwise linkage disequilibrium (LD) among all pairs of SNPs within the gene cluster. The red dotted line marks the *r*^2^=0.2. The blue dotted line represents distance where the LD drops to *r*^2^<0.2. **Figure S6.** Classification of protein family domains encoded by the gene *Zt09_13_00231.* **Figure S7.** MS scans of metabolite elution (A) Zt248 and (B) Zt231, eluting at 3.53 and 2.15 minutes, respectively. The neighboring peaks at the m/z range eluting at different retention times likely represent distinct structural isomers of the same compound.

## Data Availability

Raw information on metabolome analyses is provided as supplementary files. Genome sequencing datasets are available from the NCBI Sequence Read Archive BioProject PRJNA596434 [95] and RNA-seq datasets from BioProject PRJNA559981 (in vitro) [96] and SRP077418 (in planta) [97].
