# Supplementary material for: Genome-wide association mapping reveals genes underlying population-level metabolome diversity in a fungal crop pathogen
Source: BMC Biol. 2022 Oct 8;20:224. doi: 10.1186/s12915-022-01422-z (PMC9548119; doi:10.1186/s12915-022-01422-z)
Supplement: Supplementary file 2 — Additional file 2: Figure S1. A) The first two principal components (PC) from a PC analysis of genome-wide SNPs after removal of outliers (n=7). Isolates are color coded by the collection time point and wheat cultivar. B) SplitsTree phylogenetic network constructed from genome-wide single nucleotide polymorphism (SNP) data. Figure S2. Discriminant analysis of principal components (DAPC) results from the genome-wide SNP dataset. Cumulated variance explained by the eigenvalues of the PCs and scatter plot of the Bayesian Information Criterion (BIC) values. The lowest BIC value indicates the most parsimonious number of clusters. Figure S3. A) The first two principal components (PC) from a PC analysis of SNPs closest to biosynthetic gene clusters BGC. Isolates are color coded by the collection time point and wheat cultivar. B) Down sampling analysis to identify the proportion of 2633 metabolite markers detected in mapping population. Figure S4. Percentage leaf area covered by lesions and pycnidia during wheat infection for subset of Z. tritici strains included in the metabolome-GWAS analysis (n = 76). The isolates are grouped by their genotype at significant metabolome GWAS SNPs. Figure S5. Evolutionary history of the putative effector gene Zt09_00502. A) Alignment of the 3’UTR region of the gene. The box refers to the haplotypes found in the single Swiss field population. B) Phylogenetic tree of the 3’UTR region and (C) of the gene sequence. Names in bold black, blue and grey refer to the reference genomes of the species (IPO), isolates included in the metabolite GWAS and sister species, respectively. The phylogenetic tree was inferred by using maximum likelihood and the Tamura-Nei model. D) Pairwise linkage disequilibrium (LD) among all pairs of SNPs within the gene cluster. The red dotted line marks the r2=0.2. The blue dotted line represents distance where the LD drops to r2<0.2. Figure S6. Classification of protein family domains encoded by the gene Zt09_13_00231. F [file 12915_2022_1422_MOESM2_ESM.pdf]

Supplementary Information

Genome-wide association mapping reveals genes underlying population-level metabolome diversity in a fungal crop pathogen

Supplementary Figures

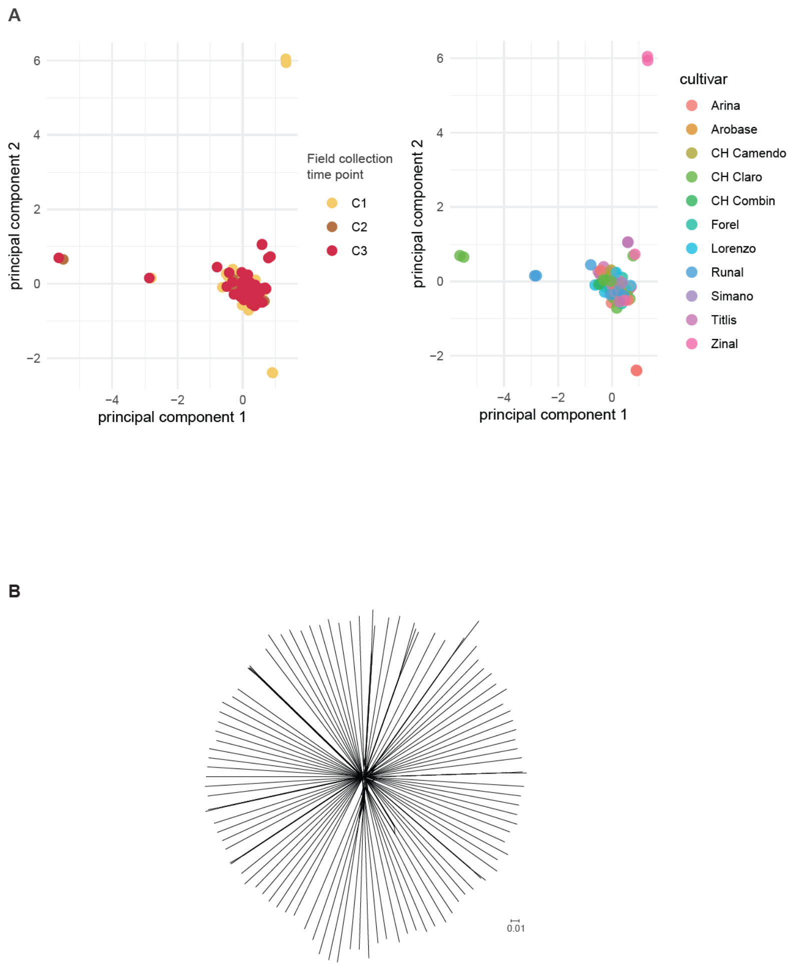

Supplementary Figure S1: A) The first two principal components (PC) from a PC analysis of genome-wide SNPs after removal of outliers (n=7). Isolates are color coded by the collection time point and wheat cultivar. B) SplitsTree phylogenetic network constructed from genome-wide single nucleotide polymorphism (SNP) data.

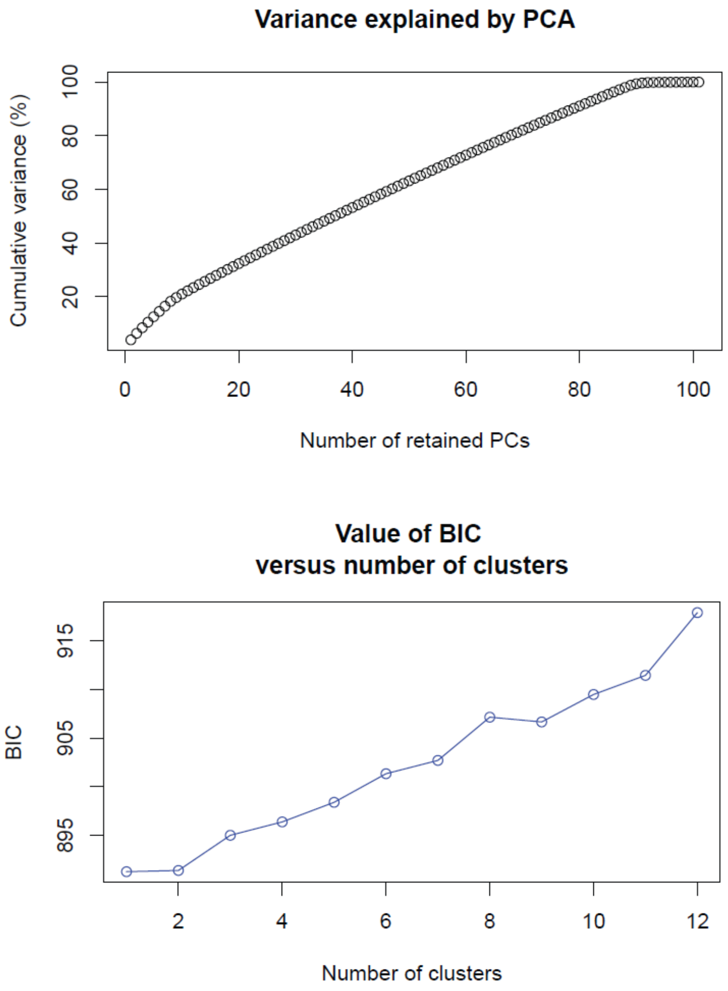

Supplementary Figure S2: Discriminant analysis of principal components (DAPC) results from the genome-wide SNP dataset. Cumulated variance explained by the eigenvalues of the PCs and scatter plot of the Bayesian Information Criterion (BIC) values. The lowest BIC value indicates the most parsimonious number of clusters.

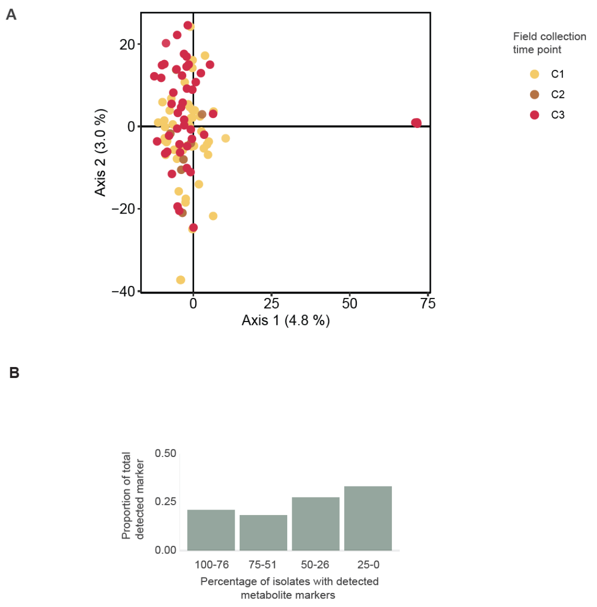

Supplementary Figure S3: A) The first two principal components (PC) from a PC analysis of SNPs closest to biosynthetic gene clusters BGC. Isolates are color coded by the collection time point and wheat cultivar. B) Down sampling analysis to identify the proportion of 2633 metabolite markers detected in mapping population.

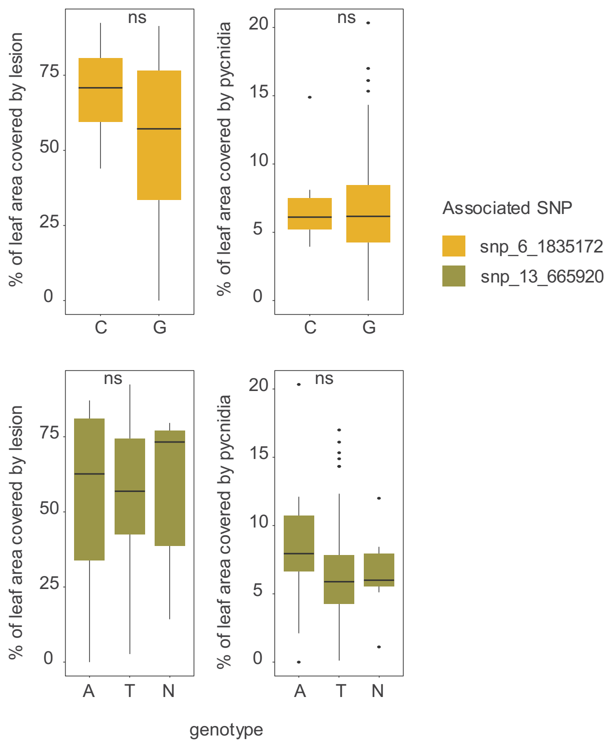

Supplementary Figure S4. Percentage leaf area covered by lesions and pycnidia during wheat infection for subset of *Z. tritici* strains included in the metabolome-GWAS analysis (*n* = 76). The isolates are grouped by their genotype at significant metabolome GWAS SNPs.

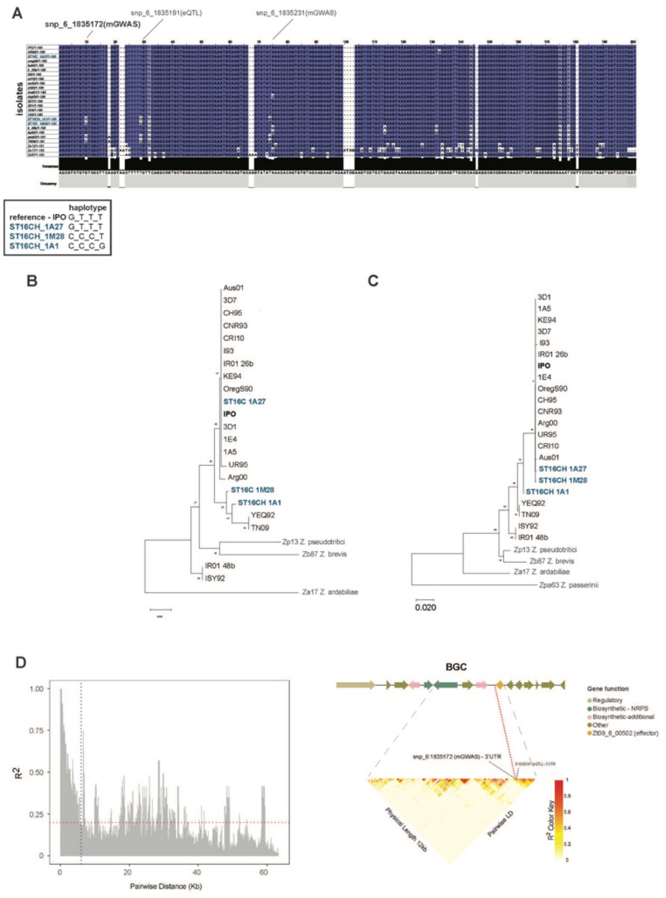

Supplementary Figure S5. Evolutionary history of the putative effector gene *Zt09_00502*. A) Alignment of the 3'UTR region of the gene. The box refers to the haplotypes found in the single Swiss field population. B) Phylogenetic tree of the 3'UTR region and (C) of the gene sequence. Names in bold black, blue and grey refer to the reference genomes of the species (IPO), isolates included in the metabolite GWAS and sister species, respectively. The phylogenetic tree was inferred by using maximum likelihood and the Tamura-Nei model. D) Pairwise linkage disequilibrium (LD) among all pairs of SNPs within the gene cluster. The red dotted line marks the r^2=0.2. The blue dotted line represents distance where the LD drops to r^2<0.2.

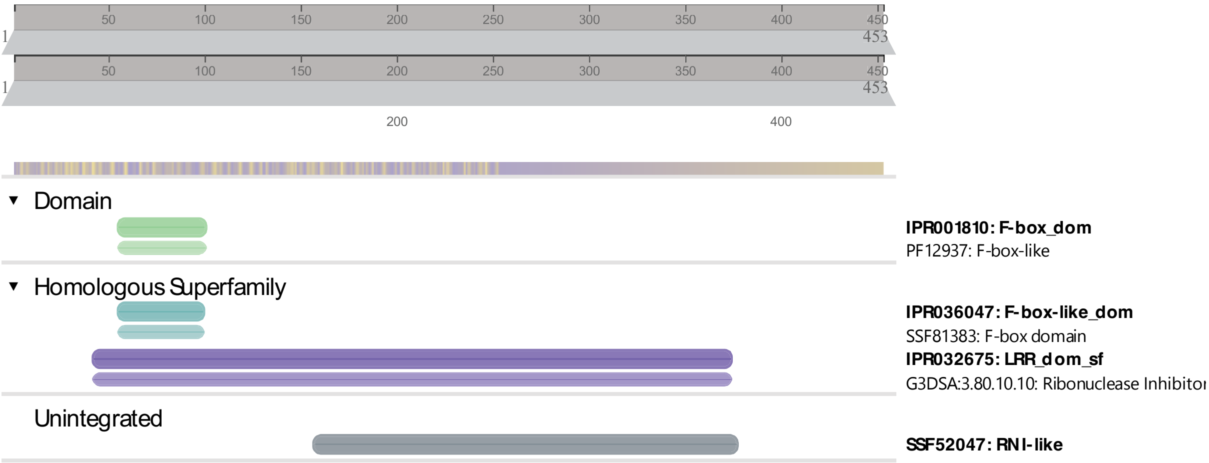

Supplementary Figure S6. Classification of protein family domains encoded by the gene *Zt09_13_00231*.

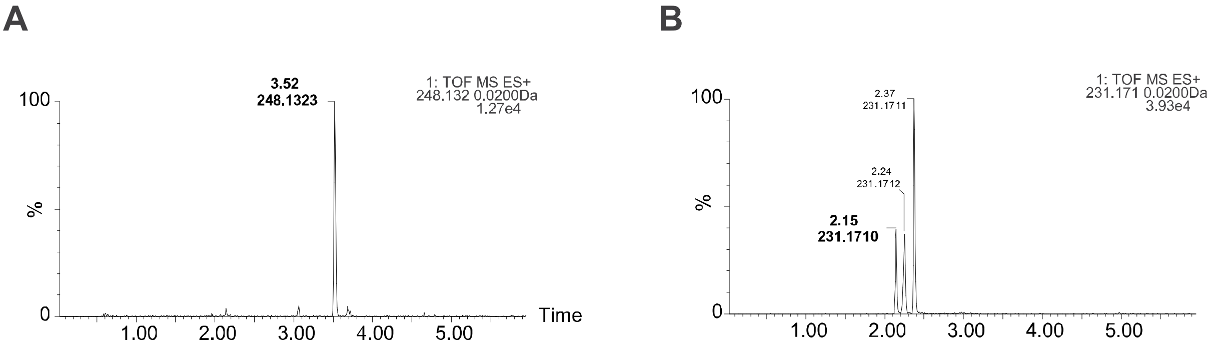

Supplementary Figure S7. MS scans of metabolite elutions (A) Zt248 and (B) Zt231, eluting at 3.53 and 2.15 minutes, respectively. The neighboring peaks at the m/z range eluting at different retention times likely represent distinct structural isomers of the same compound.
